# Monitoring the Biological Impact and Therapeutic Potential of Intermittent Fasting in Oncology: Assessing Strategies and Clinical Translational Challenges

**DOI:** 10.3390/diagnostics15182369

**Published:** 2025-09-18

**Authors:** Maria Bendykowska, Grażyna Gromadzka

**Affiliations:** 1 Faculty of Medicine, Collegium Medicum, Cardinal Stefan Wyszynski University, Woycickiego Street 1/3, 01-938 Warsaw, Poland; 2 Department of Biomedical Sciences, Faculty of Medicine, Collegium Medicum, Cardinal Stefan Wyszynski University, Woycickiego Street 1/3, 01-938 Warsaw, Poland

**Keywords:** intermittent fasting, oncology, biomarkers, immunophenotyping, autophagy, metabolic reprogramming, oxidative stress, gut microbiome, 18F-FDG PET, diagnostics, precision

## Abstract

**Background:** Intermittent fasting (IF) is emerging as a promising non-pharmacological intervention in oncology, with the potential to modulate key biological processes including metabolic reprogramming, inflammation, autophagy, and immune function, particularly through the PI3K/AKT/mTOR pathway. However, translating IF into clinical practice requires robust tools to monitor its biological impact and therapeutic effectiveness. **Objective:** This narrative review aims to present and critically evaluate current diagnostic and monitoring strategies that can support the safe and effective integration of IF into oncological care. Methods: A comprehensive literature search was conducted across PubMed/Medline, Science Direct, Scopus, Wiley Online Library, and Google Scholar using a combination of free-text and MeSH terms related to intermittent fasting, oncology, biomarkers, immunophenotyping, metabolic pathways, gut microbiome, and diagnostic imaging. **Results:** Two principal categories of monitoring objectives were identified. The first—mechanistic monitoring—focuses on elucidating IF-induced biological effects, including modulation of insulin/IGF-1 signaling, oxidative stress reduction, autophagy activation, immune reprogramming, and microbiome alterations. Advanced research tools such as single-cell RNA sequencing, proteomics, metabolomics, and circulating tumor DNA (ctDNA) assays offer high-resolution insights but currently remain limited to preclinical or translational settings due to cost and complexity. The second—clinical response monitoring—assesses IF’s impact on treatment outcomes, including chemotherapy and immunotherapy response, toxicity reduction, tumor dynamics, and maintenance of nutritional and functional status. This requires clinically validated, accessible, and interpretable diagnostic tools. **Conclusions:** A dual-layered monitoring framework that integrates both mechanistic insights and clinical applicability is essential for the personalized implementation of IF in oncology. Although preliminary findings are promising, large-scale randomized trials with standardized protocols are necessary to confirm the efficacy, safety, and feasibility of IF in routine oncological care. The integration of IF with modern diagnostics may ultimately contribute to a more individualized, biologically informed cancer treatment paradigm.

## 1. Introduction

Malignant tumors remain one of the leading causes of death worldwide, despite significant advances in oncological therapies such as targeted therapy, immunotherapy, and personalized chemotherapy regimens. The effectiveness of treatment is still often limited by the biological complexity of tumors, the resistance of cancer cells to treatment, and the systemic toxicity of therapies to normal tissues. In this context, the importance of supportive interventions that can improve treatment outcomes and the quality of life of patients is growing. Global cancer statistics 2024: GLOBOCAN estimates of incidence and mortality worldwide for 36 cancers in 185 countries [1]. One of the promising adjuvant strategies is intermittent fasting (IF), whose anticancer potential is gaining increasing recognition in light of current molecular, metabolic, and clinical data.

**Figure 1:**
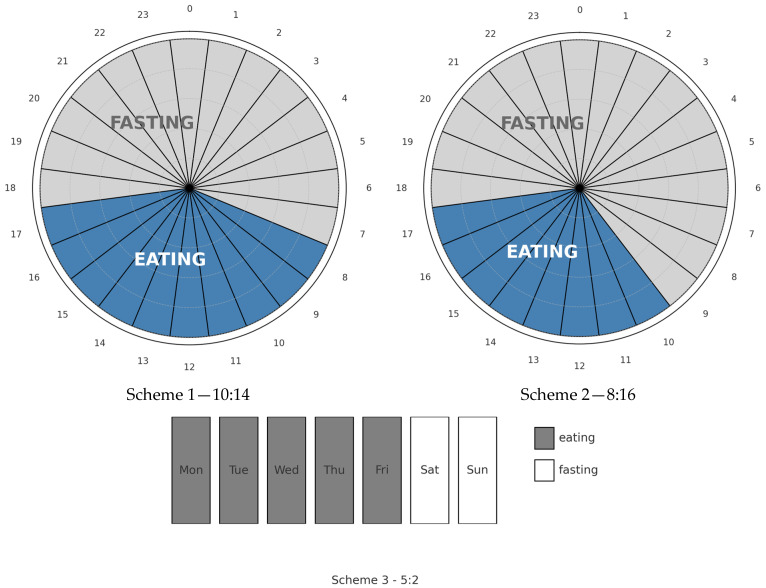
Schematic representation of intermittent fasting dietary patterns.

IF includes a range of dietary patterns based on cyclical caloric restriction—from short-term, single fasting (8–24 h) [2], to time-restricted feeding (e.g., 16/8, involving consuming all meals within an 8 h eating window with 16 h of fasting each day) [3], to longer, multi-day fasts preceding oncological treatment [4]. Examples of intermittent fasting eating patterns are shown in Figure 1. 

The mechanisms of action of IF are multifaceted and include modifications in glucose–insulin metabolism, a reduction in insulin-like growth factor 1 (IGF-1) levels, activation of autophagy, and regulation of inflammatory and immune responses. These molecular changes not only inhibit cancer cell proliferation but also contribute to the phenomenon of differential stress resistance (DSR). DSR refers to the selective protection of healthy cells and increased vulnerability of cancer cells under conditions of nutrient deprivation, enhancing the efficacy and safety of chemotherapy [5]. This differentiation in metabolic response forms the basis for the application of IF as a strategy to enhance the efficacy of anticancer therapy and reduce adverse effects. Interest in diet as a supportive element in cancer treatment is also growing among patients themselves. It is estimated that up to half of cancer patients undertake various forms of dietary interventions, often without consulting medical personnel. In this context, the need to provide reliable, evidence-based data becomes crucial for clinical practice. Clinicians require prognostic tools that enable the monitoring of the effectiveness and safety of IF as a supportive strategy—both at the level of biochemical and immunological parameters [6].

## 2. Materials and Methods

The aim of this study is to review biomarkers that can be used to monitor the safety of IF and can help assess the impact of IF on cancer treatment. Particular attention is paid to metabolic biomarkers (such as IGF-1, glucose, insulin) and immunological biomarkers (inflammatory cytokines, CRP (C-reactive protein)). Biomarkers useful in assessing the impact of IF on the severity of oxidative stress, autophagy, and the gut microbiome are also presented. This study aimed to assess the clinical usefulness of IF as a complementary treatment for oncological treatment, identify optimal methods for monitoring patients’ condition during IF, and assess the effectiveness of IF. It also identifies directions for further research on the integration of dietary strategies with modern oncology. A search for relevant articles was conducted in PubMed/Medline, Science Direct, Scopus, Willy Online Library, and Google Scholar, combining free-text and MeSH terms using a wide range of synonyms and related terms, including “intermittent fasting”; “oncology”; “biomarkers”; “immunophenotyping”; “autophagy”; “metabolic repro-gramming”; “gut microbiome”; “18F-FDG PET”; “diagnostics”; “monitoring”; “in-flammation”; “oxidative stress”; “precision medicine”, and others, as well as their combinations. Search criteria also included original research articles and review studies in humans or animal models related to cancer. This research included articles published up to May 2025.

## 3. Intermittent Fasting and Oncology

### 3.1. The Idea of Intermittent Fasting in Oncology

IF is a dietary strategy involving scheduled periods of reduced or eliminated caloric intake, interspersed with normal feeding intervals [2,3,7]. Beyond metabolic regulation and weight reduction, IF exerts systemic biological effects that align with therapeutic goals in oncology. Tumorigenesis is tightly linked to metabolic dysregulation, chronic inflammation, oxidative stress, and immune escape. IF modulates these hallmarks—improving glucose metabolism, reducing inflammation and oxidative stress, inducing autophagy, and modulating many cellular signaling pathways [3,8].

### 3.2. Molecular Mechanisms of Intermittent Fasting

IF elicits complex cellular adaptations that enhance insulin sensitivity and reduce circulating levels of glucose and IGF-1 [3,8]. These metabolic changes may impede cancer progression, as malignant cells typically exhibit elevated metabolic activity and a strong dependence on glucose as their primary energy source [8]. The reduction in insulin and IGF-1 levels can suppress key oncogenic signaling pathways, particularly the phosphoinositide 3-kinase (PI3K)/protein kinase B (AKT)/mechanistic target of rapamycin (mTOR) axis, thereby inhibiting cellular proliferation and increasing susceptibility to stress-induced apoptosis [9]. Moreover, IF induces autophagy, as indicated by biomarkers such as the lipidated form of microtubule-associated protein 1 light chain 3 (LC3-II), Beclin-1, and sequestosome 1 (p62/SQSTM1). These proteins are involved in autophagic flux, protein degradation, and intracellular signaling. Activation of autophagy may directly suppress tumor growth and contribute to the selective protection of normal cells during oncological treatment [10].

A well-characterized anticancer mechanism of IF is DSR, wherein healthy cells enter a quiescent, cytoprotective state marked by activation of DNA repair and antioxidant pathways, while cancer cells—metabolically inflexible—remain vulnerable to chemotherapy-induced cytotoxicity [5]. Clinically, this phenomenon is associated with reduced systemic inflammation, as evidenced by decreased level of CRP in breast cancer patients undergoing chemotherapy in combination with an IF regimen [11]. Preclinical studies further support the antitumor potential of short-term fasting, demonstrating decreased tumor growth rates in models of glioma, breast cancer, and melanoma, particularly when fasting is combined with chemotherapy [12,13].

### 3.3. Potential IF Impact on Cancer Therapy Tolerance and Efficacy

In addition to the cytostatic effects, IF may reduce the toxicity associated with anticancer therapies. Clinical observations and early-phase trials have demonstrated that short-term fasting—typically lasting 24 to 72 h prior to chemotherapy—can alleviate treatment-related side effects, including fatigue, nausea, mucositis, neutropenia, and cardiotoxicity [14,15,16]. Patients undergoing short-term fasting reported improved tolerance to chemotherapy, faster recovery times, and an earlier return to daily activities [4,14,15,16]. These clinical benefits are accompanied by favorable changes in inflammatory and metabolic biomarkers, such as decreased levels of IL-6 and insulin, and increased levels of circulating ketone bodies [16]. These effects are likely mediated by DSR, which selectively protects normal cells while leaving cancer cells vulnerable to cytotoxic damage.

Beyond metabolic and inflammatory modulation, IF also appears to influence the tumor immune microenvironment. Preclinical studies indicate that fasting enhances the infiltration of cytotoxic CD8^+^ T lymphocytes into tumor tissues [12] while reducing the abundance of immunosuppressive myeloid-derived suppressor cells (MDSCs) [13,17]. This immunomodulatory effect may augment the efficacy of immune checkpoint inhibitors, particularly therapies targeting the programmed cell death protein 1 (PD-1) pathway.

Together, these findings suggest that IF may not only mitigate the adverse effects of standard oncologic treatments but also improve their therapeutic efficacy by modulating both systemic metabolism and the tumor immune landscape.

### 3.4. Summary of Exisiting Clinical Evidence

Although most available data are derived from preclinical studies, several well-designed clinical trials have begun to explore the application of IF in oncology. The most compelling results to date have been observed in studies on breast cancer, where IF protocols and fasting-mimicking diets (FMDs) have been associated with improved treatment tolerability and modulation of the tumor microenvironment through immunometabolic reprogramming. In the multicenter randomized DIRECT phase II trial, patients with breast cancer receiving neoadjuvant chemotherapy alongside an FMD experienced significantly reduced toxicity and improved treatment tolerance compared to controls [15]. The study demonstrated that such dietary interventions are capable of reprogramming metabolic and immune parameters, including reductions in glucose, insulin levels, ultimately enhancing antitumor immunity [14,16].

Parallel advances are emerging in colorectal cancer. Preliminary studies suggest that IF may modulate the composition of the gut microbiota; however, human data remain limited to small observational studies. Preclinical models have shown that IF can enrich beneficial microbial populations associated with antitumor immunity and reduce microbial drivers of inflammation. These changes were accompanied by a decrease in tumor-associated macrophages and MDSCs, which are commonly implicated in immune evasion and tumor progression. Furthermore, IF promoted metabolic reprogramming in B cells, enhancing their capacity for antigen presentation and supporting a more robust antitumor immune response [18,19]. Complementing these findings, Zhong et al. (2023) reported that an FMD suppressed immunoglobulin-1 (IgA)-producing B cells, further reinforcing antitumor immunity in colorectal cancer [19]. Similarly, Luo et al. (2024) observed that an FMD improved gut barrier integrity and the immune microenvironment in colorectal cancer by modulating microbiota composition [20]. From a public health perspective, Lima Oliveira et al. (2024) proposed an IF-based intervention for colorectal cancer prevention among high-risk young adults, highlighting its preventive potential [21].

In prostate cancer, preclinical data suggest that IF mitigates age-related prostatic hyperplasia in animal models through activation of autophagic pathways and reduction of oxidative stress. These findings support the hypothesis that IF may regulate prostatic cell proliferation and inflammation through autophagy modulation [22].

In liver cancer, a study investigating Ramadan fasting revealed activation of metabolic signaling pathways, reduced systemic inflammation, and modulation of liver-resident immune cell activity, suggesting that even religious forms of fasting may exert protective immunometabolic effects within the hepatic tumor microenvironment [23].

Equally promising are findings in pancreatic cancer, a particularly aggressive malignancy. A preclinical study by Antunes et al. (2018) demonstrated that IF enhanced the uptake of a chemotherapeutic agent by increasing expression of a specific transporter protein, leading to a reduction in tumor volume by more than 40%, thus illustrating a synergistic effect between metabolic intervention and chemotherapy [10].

In summary, these findings highlight the growing interest in IF as a low-cost, biologically active intervention with the potential to support oncologic outcomes across multiple tumor types. By modulating both systemic metabolism and the local tumor microenvironment, IF may offer therapeutic benefits beyond those observed in breast cancer, with emerging evidence suggesting promise in colorectal, prostate, liver, and pancreatic malignancies.

### 3.5. Potential Risks and Safety Considerations

Despite its potential, IF carries certain risks, particularly in malnourished, cachectic, or frail patients. Prolonged caloric restriction may exacerbate sarcopenia and impair immune function. Patients with severe hepatic dysfunction, uncontrolled diabetes, or cancer-related cachexia require strict supervision due to potential metabolic instability. This vulnerability stems from the need for adequate metabolic flexibility during fasting, especially with regard to gluconeogenesis and ketogenesis, and from the risk of dangerous fluctuations in blood glucose levels, including hypoglycemia and ketoacidosis [24].

Fasting is also contraindicated during pregnancy, when both maternal and fetal metabolic demands are increased and continuous nutrient intake is essential for proper development. It is likewise unsuitable for individuals with a history of eating disorders or those with a body mass index below 18.5, due to the heightened risk of nutritional deterioration or relapse of disordered eating behaviors [8,25]. Clinical implementation of IF thus requires individualized risk–benefit assessment and careful biomarker-guided monitoring.

### 3.6. Need for Diagnostic Tools to Monitor IF Response

To ensure IF’s implementation is safe and effective, it must be monitored using appropriate monitoring biomarkers. There is a growing interest in tracking metabolic, inflammatory, and immunological responses through biomarkers such as IGF-1, CRP, IL-6, LC3, and Beclin-1, as well as assessing changes in gut microbiota composition or immune cell subsets using flow cytometry [26,27,28,29]. Advanced imaging tools like 18F-fluorodeoxyglucose positron emission tomography (18F-FDG PET) and functional magnetic resonance imaging (MRI) may serve as noninvasive methods to assess changes in tumor metabolism or perfusion. Longitudinal assessment using these modalities could help determine therapy efficacy and personalize IF protocols [30,31].

### 3.7. Benefits of Using Variable Tools in Monitoring Intermittent Fasting

The introduction of variable tools to monitor the effects of IF in oncological therapy brings a range of practical and clinical benefits. These tools not only allow for the assessment of the effectiveness of metabolic intervention but also increase the safety of the treatment by enabling better customization to the individual needs of the patient.

The use of inflammatory parameters, such as IL-6, tumor necrosis factor alpha (TNF-α), and CRP, allows for a simple and rapid evaluation of whether IF effectively reduces chronic inflammation—a factor known to be involved in the initiation and progression of cancers [32]. Regular monitoring of these parameters can support clinical decisions regarding the continuation of nutritional intervention or the need for its modification in case of risk of adverse effects from anticancer therapy. 

Equally important are metabolic tests, including the measurement of glucose, insulin, IGF-1, and insulin-like growth factor binding protein 3 (IGFBP-3) levels. A reduction in insulin axis activity and an increase in the concentration of IGF-binding proteins may indicate metabolic adaptation of the body, leading to the so-called DSR. This phenomenon supports the protection of healthy cells from chemotherapy toxicity while simultaneously increasing the sensitivity of cancer cells to treatment [4,5].

PET and MRI imaging provide non-invasive information about morphological and functional changes within the tumor. A reduction in 18F-FDG uptake (PET) following IF may indicate decreased metabolic activity of cancer cells, while perfusion and necrotic changes observed in MRI can reflect an effective tumor response to therapy supported by metabolic intervention [30,31]. 

Immunological tests, such as flow cytometry and cytokine measurements (Enzyme-Linked Immunosorbent Assay (ELISA), Luminex), allow assessment of immune system activation in response to IF [17,27]. An increase in the number of active CD8^+^ T lymphocytes and NK cells, along with a reduction in immunosuppressive MDSCs, may suggest beneficial immunomodulation supporting the anticancer response, especially in patients undergoing immunotherapy [33]. Complementing diagnostics may include analysis of the gut microbiome, which plays a key role in immune response and host metabolism. IF influences its composition and function by increasing the abundance of probiotic bacteria and the production of short-chain fatty acids (SCFAs), which may improve therapy tolerance and the patient’s clinical condition [34].

### 3.8. Monitoring Framework Theory

A structured monitoring framework is essential for the safe and effective integration of IF into oncology. This framework must begin with a clear definition of monitoring objectives, which can be broadly categorized into:

(i) Monitoring Basic Biological Effects

The aim is to investigate the mechanistic underpinnings of IF, such as:

- modulation of insulin/IGF-1 signaling,
- reduction in oxidative stress,
- activation of autophagy and stress resistance pathways,
- immune system reprogramming,
- assessment of changes in the gut microbiome.

This type of monitoring is primarily conducted in the preclinical or early translational setting and often relies on high-resolution, research-based technologies, including:

- single-cell RNA sequencing (scRNA-seq)—to examine cell-specific transcriptional responses,
- proteomics and metabolomics—to profile systemic biochemical changes,
- circulating tumor DNA (ctDNA)—to monitor early molecular dynamics.

While these tools offer invaluable insight into IF’s mechanisms of action, their complexity, cost, and turnaround time currently limit their routine clinical application.

(ii) Monitoring Clinical Treatment Response

The second objective is to assess whether IF contributes to clinical benefits or safety risks, such as:

- improved response to chemotherapy or immunotherapy,
- reduced treatment-related toxicities,
- stabilization or regression of tumor burden,
- preservation of nutritional and functional status.

This requires clinically applicable tools that are validated, accessible, and interpretable in real time, including:

- CRP and IL-6—systemic inflammation markers,
- fasting glucose, insulin, and ketone levels—metabolic status indicators,
- albumin and prealbumin—nutritional status markers,
- complete blood count (CBC)—hematologic safety,
- PET/ computed tomography (CT) imaging—to monitor changes in tumor metabolic activity and response.

Importantly, the integration of both monitoring layers—mechanistic insight and clinical applicability—enables a more translationally relevant and patient-centered approach to IF research and implementation.

## 4. Monitoring the Effectiveness of Intermittent Fasting

### 4.1. Monitoring Basic Biological Effects

#### 4.1.1. Modulation of Insulin/IGF-1 Signaling

One of the principal metabolic pathways modulated by IF is the insulin/IGF-1 axis. This signaling cascade plays a central role in the regulation of cellular growth, proliferation, and survival and is frequently dysregulated in various malignancies. Elevated circulating levels of insulin and IGF-1 have been associated with increased tumorigenesis in several types of cancer, including breast, prostate, and colorectal cancers [35,36].

IF has been shown to reduce circulating concentrations of insulin and IGF-1, thereby attenuating activation of the downstream PI3K/Akt/mTOR pathway, which is critically implicated in oncogenic processes [9]. These metabolic alterations resemble those induced by caloric restriction and are associated with reduced cellular proliferation, increased chemosensitivity of malignant cells, and enhanced stress resistance mechanisms in normal cells [8].

Modulation of this pathway can be monitored through measurement of fasting serum insulin, IGF-1, and IGFBP-3 levels, as well as by evaluating insulin resistance indices, such as the homeostatic model assessment for insulin resistance (HOMA-IR). In preclinical models, short-term fasting has led to reductions in IGF-1 levels of up to 50% within 48–72 h [37] comparable trends reported in early-phase clinical trials [14].

Accordingly, biomarkers related to insulin/IGF-1 signaling may serve not only as indicators of IF efficacy but also as potential tools for treatment stratification and personalization.

#### 4.1.2. Reduction in Oxidative Stress

Oxidative stress, defined as an imbalance between the production of reactive oxygen species (ROS) and the capacity of antioxidant defense mechanisms, represents a critical factor in cancer initiation and progression. Malignant cells frequently exhibit elevated ROS levels, which contribute to DNA damage, genomic instability, and the promotion of angiogenesis. However, excessive oxidative stress may also render cancer cells susceptible to redox-modulating therapeutic strategies.

IF has been associated with a reduction in oxidative stress, partly through the enhancement of mitochondrial function and the upregulation of endogenous antioxidant defenses, including superoxide dismutase and catalase. Simultaneously, IF appears to suppress pro-oxidant pathways, thereby promoting a more favorable redox balance. These effects are thought to be mediated by metabolic sensors and stress-response regulators such as AMP-activated protein kinase and nuclear factor erythroid 2-related factor 2 (Nrf2 or NFE2L2), which together facilitate cellular adaptation to metabolic challenges [38].

To assess these effects, studies frequently utilize biomarkers of oxidative damage and antioxidant capacity. Commonly measured indicators include (1) circulating levels of parameters of oxidative damage to various cellular structures: (i) plasma prostaglandin F2α 8-epimer (8-iso-PGF2α) as a strong marker of oxidative damage to cell membranes; (ii) plasma 3-nitrotyrosine, as a marker of the oxidative modification of proteins; (iii) saliva 8-hydroxy-2′deoxyguanosine (8-OHdG), a product of oxidative DNA damage; (2) activity of small-molecule (e.g., glutathione, GSH) and large-molecule enzymatic antioxidants (for example, catalase (Cat), manganese-dependent superoxide dismutase (MnSOD), or glutathione peroxidase (GPx)) in peripheral blood cells; as well as (3) total antioxidant capacity (TAC). Across multiple investigations, IF protocols have been consistently associated with reductions in these markers, reflecting a systemic decrease in oxidative stress [38].

Notably, IF may exert a dual effect: conferring protection to healthy tissues by attenuating oxidative damage while concurrently increasing oxidative pressure within cancer cells—a condition that may sensitize them to therapeutic interventions such as chemotherapy or radiotherapy. Accordingly, monitoring oxidative stress dynamics during IF may provide mechanistic insights and support the optimization of individualized oncologic treatment strategies.

#### 4.1.3. Analysis of Activation of Autophagy and Stress Resistance Pathways

Autophagy, a tightly regulated process of cellular component degradation, plays a critical role in maintaining cellular homeostasis and conferring resistance to stress. In oncology, autophagy exhibits a dual role: while it may promote tumor cell survival under stress conditions, excessive activation can lead to tumor cell death. Its role in carcinogenesis is context-dependent and varies according to the stage of tumor development [39]. In early carcinogenesis, autophagy exerts a protective function by eliminating damaged organelles and genetically unstable cells, thereby potentially preventing malignant transformation. In contrast, in advanced malignancies, autophagy often serves as a survival mechanism that enables tumor cells to adapt to metabolic stress, hypoxia, and chemotherapy. Therefore, modulation of autophagy in cancer therapy must be carefully tailored to the tumor type and disease stage to avoid inadvertently facilitating tumor progression [40,41].

In the oncological setting, autophagy activation induced by IF may act synergistically with anticancer therapies, particularly chemotherapy and immunotherapy.

IF also promotes autophagy through inhibition of the mTOR pathway and activation of AMP-activated protein kinase. In a study by Alirezaei et al. (2010), a 24 h fast led to increased levels of LC3, a widely recognized marker of autophagy activation, in murine neurons [42] Similarly, Madeo et al. (2015) reported that IF increased Beclin-1 expression and the conversion of LC3-I to LC3-II, indicating enhanced autophagosome formation. Beclin-1 initiates autophagy, while LC3-II, generated through lipidation and incorporation into the autophagosomal membrane, is considered a key molecular indicator of active autophagy. Activation of autophagy by IF may facilitate the removal of damaged cellular components, supporting normal cell survival while impairing cancer cells, which often exhibit defective regulation of this process [43].

Enhanced autophagy has been shown to increase the efficacy of chemotherapy and to promote immunogenic cell death in malignant cells [44]. An increase in LC3-II levels following IF may therefore serve as a biomarker indicating enhanced tumor susceptibility to therapy [43].

Nevertheless, clinical application of LC3 as a biomarker remains limited. Tumor biopsies rarely allow for standardized quantification of LC3 due to restricted tissue accessibility and variability. Moreover, the relationship between LC3-II levels in peripheral blood and intratumoral autophagic activity has not been conclusively established, limiting its utility in routine clinical settings [45]. 

The interpretation of autophagy biomarkers such as LC3 and Beclin-1 presents additional challenges. For example, elevated LC3 levels may reflect either enhanced autophagy or impaired autophagic flux. Beclin-1 expression also varies across tumor types and conditions, which constrains its generalizability as a biomarker in cancer patients undergoing IF [41,43].

Assessment of autophagy typically involves immunoblotting for markers such as LC3, Beclin-1, and p62 (Sequestosome-1), confocal microscopy for autophagosome visualization, and flow cytometry using fluorescent dyes such as Cyto-ID. Recently, in vivo imaging techniques employing fluorescent probes and two-photon microscopy have been developed to monitor autophagy dynamics in real time [46].

#### 4.1.4. Immune System Reprogramming

IF influences numerous components of the immune system, affecting both innate and adaptive responses, thereby modulating the potential effectiveness of anticancer therapies. Preclinical studies have demonstrated activation of cytotoxic CD8^+^ T lymphocytes, an increase in natural killer (NK) cells, as well as a reduction in immunosuppressive MDSCs. Such immunomodulation may enhance the antitumor response and improve control over tumor progression. In a mouse model, IF was shown to increase the expression of interferon gamma (IFN-γ) and perforin in CD8^+^ T cells, indicating enhanced cytotoxic activity. Simultaneously, a reduction in the expression of the PD-1 receptor was observed, whose overexpression is associated with T cell “exhaustion”—a phenomenon limiting the effectiveness of immunotherapy. The impact of IF on this mechanism suggests its potential use as a supportive strategy in therapies involving immune checkpoint inhibitors [12,17,47].

In clinical settings, assessing the impact of IF on the immune system requires the use of advanced monitoring biomarkers. The most used techniques include flow cytometry—for quantitative analysis of T lymphocyte subpopulations (CD4^+^, CD8^+^, Treg, Th1, Th17), B cells, natural killer (NK) cells, and MDSCs [27,48]. It is also possible to evaluate the expression of regulatory molecules. NK cell functional tests—cytotoxicity assays or degranulation assays [49] ELISA/Luminex—measurement of cytokine and chemokine levels in patient serum, e.g., IFN-γ, IL-2, IL-10, TNF-α [50]. Immunophenotyping techniques such as flow cytometry and ELISA have become increasingly standardized and integrated into clinical practice. These methods are now widely incorporated into diagnostic and prognostic workflows in oncology. Nevertheless, successful implementation still requires strict adherence to standardized protocols, careful fluorochrome panel design, and trained personnel to ensure reliable and reproducible results [27,48,49].

In the future, immunological biomarkers may become an integral part of personalized oncology therapy involving IF. Monitoring these biomarkers before, during, and after treatment could not only provide information on the biological efficacy of the intervention but also help predict treatment response and risk of complications.

#### 4.1.5. Assessing MDSC Dynamics in Response to Intermittent Fasting

Given the emerging role of IF in modulating immune responses in cancer, immunophenotyping of peripheral blood cells has gained relevance as a translational monitoring tool. MDSCs, known for their immunosuppressive activity and tumor-promoting functions, represent a particularly relevant immune cell population affected by metabolic interventions.

Flow cytometry-based identification and quantification of MDSC subpopulations provides a feasible and informative strategy for evaluating the immunomodulatory effects of IF in both preclinical and early clinical settings [48].

A recommended flow cytometry panel to assess monocytic and granulocytic MDSCs in humans should include the following markers:

- CD33^+^ (cluster of differentiation 33^+^) HLA-DR^−^ (Human Leukocyte Antigen—DR isotype)/low—core phenotype of MDSCs,
- CD11b, CD14, CD15—to differentiate monocytic (M-MDSC) from granulocytic (PMN-MDSC; Polymorphonuclear MDSCs) subsets,
- lineage exclusion markers (e.g., CD3, CD19, CD56)—to improve specificity by gating out lymphoid populations,
- viability dye—to ensure accurate discrimination of live cells.

This panel enables the identification of:

- M-MDSCs: CD11b^+^ CD14^+^ HLA-DR^−^/low CD15^−^,
- PMN-MDSCs: CD11b^+^ CD15^+^ CD14^−^ HLA-DR^−^/low.

Longitudinal analysis of circulating MDSCs before and after IF cycles may help evaluate immunological shifts associated with fasting regimens and predict treatment responsiveness, particularly in patients receiving immunotherapy.

Importantly, MDSC monitoring can be implemented using peripheral blood mononuclear cells (PBMCs) and standardized gating strategies, making it suitable for early-phase clinical trials and mechanistic studies.

Further integration of functional assays (e.g., arginase activity, ROS production) and cytokine profiling (e.g., IL-6, granulocyte-macrophage colony-stimulating factor [GM-CSF]) may complement the phenotypic characterization of MDSC activity and provide a more comprehensive picture of IF-induced immune modulation.

Emerging preclinical and early clinical studies support the relevance of MDSC monitoring in the context of IF in oncology:

Preclinical evidence (murine 4T1/4T07 breast cancer models) demonstrates that IF reduces splenic accumulation of granulocytic MDSCs (CD11b^+^ CD33^+^ HLA-DR^−^/low CD15^+^) via CXCR4 (C-X-C chemokine receptor type 4) downregulation and metabolic stress-induced apoptosis [18].

Clinical pilot data from fasting-mimicking diet trials in cancer patients (e.g., breast and prostate cancer, phase I–II) report decreased circulating levels of MDSC-related cytokines (IL-6, chemokine (C-C motif) ligand 2 [CCL2], granulocyte colony-stimulating factor [G-CSF]), suggesting indirect modulation of MDSC expansion [17]. 

These findings support the utility of incorporating MDSC profiling into IF studies using flow cytometry panels tailored to detect both monocytic and granulocytic subsets in translational settings. A brief summary of the preclinical evidence regarding IF-related MDSC modulation is presented in Table 1.

#### 4.1.6. Assessment of Changes in the Gut Microbiome

The gut microbiota plays a crucial role in regulating immunity, metabolism, and response to anticancer therapy. Increasing evidence indicates that its composition can influence the efficacy of immunotherapy and modulate chronic inflammation that promotes tumorigenesis. Altered timing of nutrient intake—and thus circadian rhythm of digestion—affects gut bacterial activity and the production of beneficial metabolites, such as short-chain fatty acids like butyrate, which maintain intestinal epithelial integrity and suppress pro-inflammatory signaling pathways (e.g., NF-κB—Nuclear Factor kappa-light-chain-enhancer of activated B cells) [20]. Preclinical studies suggest that IF contributes to an increase in populations of anti-inflammatory bacteria that support mucosal barrier function and improve insulin sensitivity—such as *Akkermansia muciniphila* and *Lactobacillus* spp.—while reducing the abundance of pro-inflammatory Enterobacteriaceae [20]. These changes may enhance the effectiveness of anticancer therapies—including immunotherapy—as demonstrated in melanoma patients, where microbiome diversity correlated with a better treatment response. Their microbiomes showed a predominance of bacteria from the *Ruminococcaceae* family [29]. Analysis of microbiota is mainly based on 16S rRNA sequencing and shotgun metagenomics, while metabolites are measured using chromatography and mass spectrometry. Gut microbiota analysis via 16S rRNA sequencing offers only genus-level resolution and provides limited insight into the functional capabilities of microbial communities. Furthermore, the microbiome is highly dynamic and influenced by numerous external variables, including diet, antibiotics, and sample handling procedures, which makes consistent interpretation difficult [20,34].

### 4.2. Monitoring Clinical Treatment Response

#### 4.2.1. Systemic Inflammation Markers

Chronic inflammation is a characteristic feature of many cancers and plays a crucial role in carcinogenesis, tumor progression, and metastasis. Pro-inflammatory cytokines, such as IL-6, tumor necrosis factor-alpha (TNF-α), and CRP, serve not only as biomarkers of inflammation but also actively contribute to promoting a tumor-supportive microenvironment [51]. 

However, not all studies confirm a definitive effect of IF on inflammatory markers. A systematic review conducted by Cienfuegos et al. (2023) indicates that the effects of IF on IL-6 and TNF-α levels are inconclusive, highlighting the need for further research in this area [52].

Measurement methods typically include enzyme-linked immunosorbent assay (ELISA) techniques for quantitative analysis of cytokines in blood serum, as well as multiplex technologies (e.g., Luminex), which allow simultaneous measurement of multiple inflammatory markers. Monitoring the dynamics of changes in these biomarker concentrations can serve as a tool to evaluate the effectiveness of fasting in the immunomodulatory and anticancer context. Despite their widespread use, inflammatory biomarkers such as CRP, IL-6, and TNF-α suffer from limited specificity. These markers can be elevated due to various non-malignant conditions, including infections or autoimmune disorders, which reduce their diagnostic accuracy in oncology. In addition, many cytokines have a short half-life, making them highly sensitive to the timing of blood collection. Without standardized sampling times, fluctuations due to circadian rhythms or treatment-related stress may confound results. Moreover, these markers show significant inter-individual variability, making longitudinal tracking challenging [53,54].

#### 4.2.2. Metabolic Studies and Lipid Profile

Metabolic disorders such as hyperinsulinemia, insulin resistance, and dyslipidemia are frequently observed not only as lifestyle-related conditions but also among cancer patients, where they are associated with poorer prognosis. IF positively influences metabolic parameters by reducing blood glucose and insulin levels, enhancing insulin sensitivity, and lowering circulating concentrations of IGF-1 [55]. Clinical studies have demonstrated that even short-term time-restricted feeding regimens (e.g., 16/8 or 20/4—referring to an 8 or 4 h feeding window followed by 16 or 20 h of fasting) can significantly improve glycemic control and lipid profiles.

Sutton et al. (2018) reported improvements in HOMA-IR as well as reductions in fasting insulin and triglyceride levels in patients with insulin resistance without concomitant weight loss [3]. Similarly, Tinsley and La Bounty (2015) highlighted that IF may increase high-density lipoprotein (HDL) cholesterol and decrease low-density lipoprotein (LDL) cholesterol, thereby potentially reducing the risk of atherosclerosis and inflammation-related carcinogenic processes [2].

Monitoring of metabolic changes typically includes fasting glucose measurements, oral glucose tolerance tests (OGTTs), glycated hemoglobin (HbA1c), and a comprehensive lipid panel (LDL, HDL, triglycerides, and total cholesterol). In the oncological context, concurrent assessment of IGF-1 and its primary binding protein IGFBP-1 provides additional insight into the activity of the IGF axis—a key regulator of carcinogenesis [37] 

The IGF axis plays a critical role in regulating cellular growth, differentiation, and survival. Its excessive activation promotes cancer cell proliferation, inhibits apoptosis, and increases the risk of malignant transformation—particularly in hormone-dependent tumors such as breast, prostate, and colorectal cancers [56,57]. 

IF has been shown to modulate this signaling pathway by decreasing circulating IGF-1 levels, leading to downregulation of the PI3K/AKT/mTOR pathway, a central driver of tumor growth. Simultaneously, IF increases the levels of IGFBP-1, which binds IGF-1 and reduces its bioavailability, thereby attenuating pro-proliferative signaling [58,59]. 

In human studies, adherence to an FMD for five days per month over three consecutive cycles resulted in a significant reduction in IGF-1 levels. These changes were associated with decreased inflammatory markers and improved metabolic parameters [37]. Thus, by modulating the IGF-1/IGFBP-3 axis, IF may lower cancer risk and enhance the effectiveness of anticancer therapies through inhibition of growth-promoting signals in tumor cells.

Despite their utility, metabolic markers such as glucose, insulin, and IGF-1 are highly sensitive to external factors, including food intake, physical activity, circadian rhythms, and psychological stress. Moreover, the lack of standardization in IGF-1 assays complicates cross-study comparisons. Short-term fluctuations in these biomarkers may also fail to capture long-term metabolic remodeling, limiting their interpretability in clinical and research settings [60]. 

#### 4.2.3. Albumin and Prealbumin—Nutritional Status Markers

Albumin and prealbumin are among the most commonly used laboratory indicators of nutritional status, which is particularly important in cancer patients undergoing meta-bolic interventions such as IF. Albumin, synthesized in the liver, has a relatively long half-life (around 21 days) and is generally considered a marker of chronic malnutrition. In contrast, prealbumin—also known as transthyretin—with a much shorter half-life (around 2 days), is more sensitive to acute changes in nutritional status.

In several clinical studies assessing IF or FMD in oncology, albumin and prealbumin were monitored as indicators of nutritional safety. For example, Vernieri et al. (2022) re-ported that patients undergoing FMD did not exhibit significant reductions in albumin levels, suggesting metabolic tolerance and safety of short-term fasting [17]. Similarly, de Groot et al. (2020) found no clinically relevant decreases in albumin or prealbumin levels in breast cancer patients undergoing chemotherapy and IF, reinforcing the feasibility of such interventions without compromising nutritional status [15].

Thus, monitoring these markers serves a dual purpose: as markers of potential mal-nutrition risk in overly restrictive IF protocols, and as tools for assessing patient tolerance and adaptation to metabolic interventions.

#### 4.2.4. Complete Blood Count—Hematologic Safety

Complete blood count (CBC) is a routine and essential test used to evaluate hematologic safety in cancer patients, particularly when introducing dietary or metabolic interventions. It includes evaluation of white blood cells (WBCs), red blood cells (RBCs), hemoglobin, hematocrit, platelets, and indices such as mean corpuscular volume (MCV) and red cell distribution width (RDW).

In the context of IF, particular attention is paid to WBC counts—especially neutrophils and platelets, as these parameters can indicate immunosuppression or treatment-related toxicity.

Studies have explored the hematologic effects of IF. Safdie et al. (2009) reported that short-term fasting in patients undergoing chemotherapy may reduce myelosuppression and help preserve hematopoietic function. These findings were supported by subsequent research suggesting that IF may induce a DSR response, in which healthy hematopoietic cells enter a protective metabolic state, reducing susceptibility to chemotherapy-induced damage [4].

However, in patients with preexisting cytopenia or those receiving intensive myelosuppressive therapy, IF protocols should be implemented with caution. Regular CBC monitoring is crucial to ensure safety and detect any decline in hematologic parameters [4].

#### 4.2.5. Imaging Using PET and MRI

IF modulates several key biological processes in cancer cells, including energy metabolism, glucose uptake, angiogenesis, and proliferative signaling pathways. These effects create opportunities for evaluating IF-induced changes using advanced imaging modalities. Particularly relevant are positron emission tomography (PET) with the radiotracer 18F-FDG and MRI incorporating functional sequences that assess the tumor microenvironment [26,30].

18F-FDG PET enables the assessment of tumor metabolic activity by quantifying glucose uptake. Due to the Warburg effect, cancer cells demonstrate elevated glucose consumption and overexpression of glucose transporters, such as glucose transporter type 1 (GLUT1). In the study by Weng et al. (2020), short-term fasting was shown to downregulate the expression of GLUT1, mTOR, and hypoxia-inducible factor 1-alpha (HIF-1α), leading to decreased FDG uptake in tumor cells. MicroPET/CT imaging confirmed a significant reduction in standardized uptake value (SUV) in mice with colorectal cancer following dietary restriction, indicating suppressed glycolytic activity and suggesting a favorable therapeutic effect of IF [26].

MRI, especially diffusion-weighted imaging (DWI) and dynamic contrast-enhanced MRI (DCE-MRI), provides detailed insights into tumor morphology and microenvironmental features, including necrosis, hypoxia, and vascular permeability. These modalities enable the evaluation of IF-induced effects by detecting alterations in tumor perfusion or tissue response to reduced energy availability [30]. In a review by Yang et al. (2022), the significance of tumor metabolic reprogramming as a component of imaging diagnostics was underscored. The authors emphasized that reduced glucose availability, a consequence of IF, results in decreased metabolic activity as visualized on PET, while the expression of GLUT1 and HIF-1α may serve as molecular correlates of the observed imaging changes [61]. 

Thus, imaging techniques such as PET and MRI, when performed before and after IF interventions, may serve as objective tools to assess the efficacy of adjuvant therapies aimed at modulating tumor metabolism.

Despite their diagnostic utility, both PET and MRI have notable limitations. PET with 18F-FDG may yield false-positive results due to elevated glucose uptake in non-malignant processes such as inflammation or infection. Furthermore, its sensitivity varies by tumor type, potentially underestimating lesions with low glycolytic activity. Advanced MRI techniques such as DWI and DCE-MRI require cross-platform standardization and are susceptible to motion artifacts and operator-dependent variability. Additionally, both PET and MRI are resource-intensive and may not be universally accessible in routine clinical practice [26,30].

A comparative overview of monitoring tools used to evaluate the effects of intermittent fasting in oncology is presented in Table 2.

## 5. Clinical Decision Pathways

**Figure 2:**
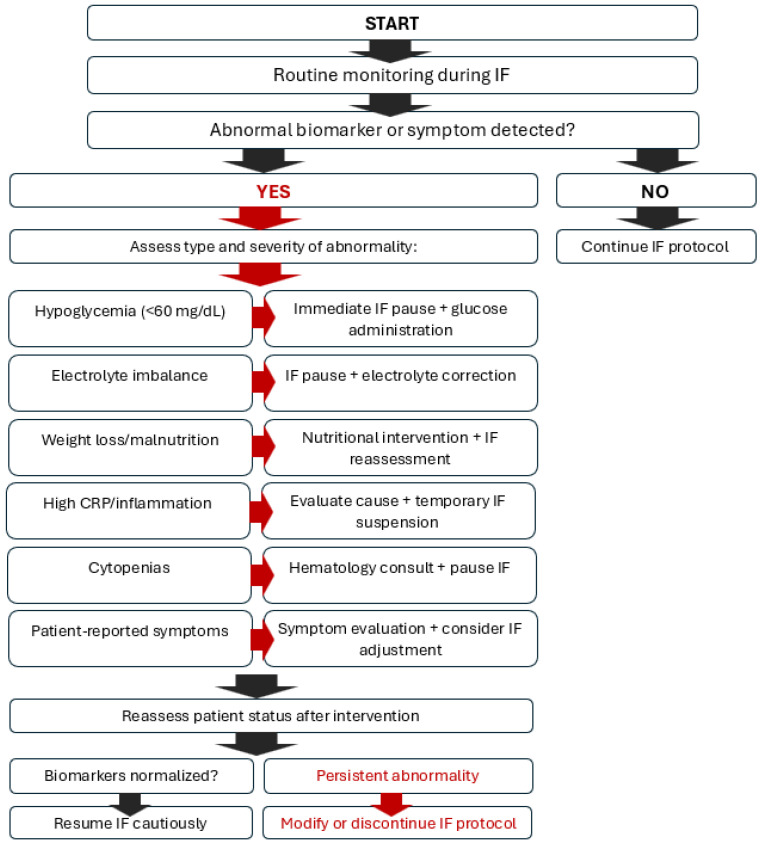
Clinical decision flowchart for managing abnormal biomarker values during intermittent fasting in oncology patients.

To ensure patient safety and optimize therapeutic outcomes, IF regimens in oncology must be guided by dynamic clinical monitoring and decision-making pathways. Specific biomarker changes may necessitate a temporary pause, dose adjustment, or discontinuation of IF protocols. A clinical decision algorithm integrating key metabolic, hematological, and nutritional parameters is presented in Figure 2.

Key triggers for intervention include:

- Severe hypoglycemia (e.g., blood glucose < 60 mg/dL): immediate IF discontinuation and nutritional intervention.
- Electrolyte imbalances (e.g., sodium < 130 or >150 mmol/L; potassium < 3.0 or >5.5 mmol/L): temporary IF pause; correction of electrolyte status; re-evaluation before resumption.
- Excessive weight loss or malnutrition (e.g., >5% body weight loss in <2 weeks; albumin < 3.0 g/dL): nutritional support, potential IF protocol modification.
- Elevated inflammatory markers (e.g., CRP > 3× baseline): consider treatment-related toxicity or infection; IF pause until clinical resolution.
- Cytopenias (e.g., absolute neutrophil count [ANC] < 1000/μL, platelets < 50,000/μL): assessment of bone marrow function and therapy impact; IF pause until recovery.
- Patient-reported symptoms (e.g., dizziness, syncope, fatigue): symptom-driven IF interruption; individualized reassessment.

The algorithm promotes a personalized and responsive approach, incorporating clinical judgment, patient preferences, and evolving evidence. It underscores the importance of a multidisciplinary team, including oncologists, dietitians, and clinical researchers, in safely integrating IF into cancer care.

## 6. Technical Limitations

Despite promising findings regarding the potential role of IF as an adjunct to oncological therapies, several technical limitations and challenges must be acknowledged when interpreting results and considering clinical implementation.

First, methodological limitations may affect the accuracy of assessing IF-related effects. For instance, 16S rRNA gene sequencing, commonly used to analyze gut microbiota composition, lacks the resolution to distinguish functional capabilities of bacterial strains, thereby limiting comprehensive understanding of the microbiological mechanisms underpinning IF-induced effects. While metagenomic and metatranscriptomic approaches offer greater functional insight, they remain costly, data-intensive, and are not yet widely standardized for clinical use [52].

Furthermore, the correlation between false-positive rates in 18F-FDG PET imaging and tumor type remains insufficiently characterized. Non-malignant processes such as inflammation, infection, and tissue repair can lead to increased glucose uptake and thus contribute to diagnostic ambiguity. This complicates the interpretation of PET scans in the context of IF, where metabolic shifts may occur both in cancerous and non-cancerous tissues [3,53].

Another significant limitation is the heterogeneity of IF protocols employed across studies. Variations in fasting duration, caloric restriction intensity, and feeding window (ranging from time-restricted feeding of 8–24 h to periodic fasting over several days) may result in diverse biological responses, impeding comparability and the establishment of standardized clinical recommendations [2]. Additionally, patient-specific factors such as age, sex, tumor type, and metabolic status can further modulate responses to IF interventions. From a clinical perspective, IF carries potential risks, particularly in vulnerable populations such as individuals with malnutrition, cancer-associated cachexia, uncontrolled metabolic disorders, or comorbidities that may be exacerbated by caloric restriction. In these cases, IF may lead to deterioration in clinical status, immune function, or treatment tolerance, underscoring the importance of careful patient selection and medical supervision [25].

Finally, the lack of validated, standardized monitoring tools and biomarkers hampers the routine clinical integration of IF. Although advanced imaging modalities such as PET and MRI, as well as metabolic and immunological biomarker profiling, hold promise for monitoring treatment response and safety, these approaches require further validation in large-scale clinical trials [53,54].

For these reasons, both researchers and clinicians should interpret current evidence with caution. Future efforts should focus on the standardization of IF protocols, development of functional microbiome assays, and validation of dynamic biomarkers capable of capturing IF-induced metabolic, immunological, and oncological changes in a reliable and reproducible manner.

## 7. Summary

IF represents a promising adjunctive strategy in oncological treatment, modulating a range of critical biological mechanisms—including glucose metabolism, immune function, inflammatory processes, and autophagy. In the context of cancer therapy, IF has demonstrated potential not only in limiting tumor growth and proliferation but also in reducing the toxicity associated with anticancer treatments, as evidenced by preclinical studies and preliminary clinical trials. Potential benefits, risks, and applications of IF in oncology are presented in Table 3.

The implementation of advanced monitoring tools—such as assays for inflammatory and metabolic markers, immunological profiling, microbiome analysis, and functional imaging—facilitates precise and dynamic assessment of IF’s effects on cancer progression. These technologies enable both the objective evaluation of therapeutic efficacy and the adaptation of fasting protocols to an individual patient’s biological response.

From a clinical standpoint, IF may enhance treatment tolerance, mitigate adverse effects, support the efficacy of immunotherapy, and improve overall quality of life. Emerging evidence also highlights its positive influence on patients’ subjective well-being, an important aspect of psychological support during cancer care. A comparative summary of IF’s potential benefits and risks in oncology is presented in Table 3.

Importantly, the practical implementation of IF in oncological settings requires careful integration with personalized nutritional counseling, continuous medical supervision, and biomarker-driven monitoring. A structured monitoring framework—incorporating dynamic parameters such as cytokine levels, immune profiles, metabolic indicators, and advanced imaging—forms the basis for real-time, adaptive modulation of therapy guided by biological feedback. This approach, combining mechanistic insights with clinically actionable biomarkers, is crucial for the safe and effective clinical integration of IF.

Despite encouraging preliminary results, the routine clinical application of IF still necessitates validation through large-scale, multicenter, randomized controlled trials with standardized protocols and rigorous safety assessments. Further research aimed at optimizing fasting regimens and combining them with specific therapeutic modalities will be essential for incorporating IF into evidence-based, personalized oncology. Ultimately, the integration of IF with modern diagnostic tools and anticancer therapies may become a key component of comprehensive, patient-centered cancer treatment.

## Figures and Tables

**Table 1:** Preclinical evidence: IF/ FMD and MDSC modulation.

Model/Study	MDSC Effect of IF
4T1/4T07 murine breast cancer + IF	↓ Splenic CD205^+^ G-MDSCs via CXCR4 downregulation & apoptosis
FMD/IF in murine 4T1 model	↓ Tumor MDSCs, ↑ T cell ratio in PBMCs
Human FMD cancer patients	↓ Circulating cytokines (IL-6, CCL2, G-CSF) that mobilize MDSCs

CCL2, chemokine (C-C motif) ligand; CXCR4, C-X-C chemokine receptor type; FMD, Fasting-Mimicking Diets; IF, G-CSF, Granulocyte colony-stimulating factor; IL-6, interleukin-6; Intermittent Fasting; MDSC, Myeloid-derived suppressor cells; PBMC, peripheral blood mononuclear cells; ↑ indicates downregulation, ↓ indicates upregulation.

**Table 2:** Comparative overview of selected monitoring tools used to evaluate the effects of intermittent fasting in oncology.

Method	Technique	Benefit	Limitations
**Inflammatory markers** **(IL-6, CRP, TNF-α** **)**	ELISA, Luminex	Assessment of inflammation and tumor microenvironment, ELISA: Sensitivity: high but depends on the specific kit; Specificity: very good, monoclonal antibodies minimize cross-reactivity Luminex: Sensitivity: comparable or higher than ELISA but can be lower for some analytes; Specificity: good but risk of cross-reactivity between antibodies in the multiplex panel	ELISA: single analyte per sample, time consuming, requires larger sample volume Luminex: higher cost of equipment and reagents, requires calibration, possible multiplex interference In summary: ELISA is more specific and simpler but analyzes only one marker at a time; Luminex allows simultaneous measurement of multiple cytokines/CRP from less sample but with potential lower specificity and higher cost
**Metabolic profile** **(glucose, insulin, IGF-1, IGFBP-3)**	OGTT, HOMA-IR, chemiluminescence	Evaluation of glucose–insulin homeostasis and IGF-1 axis	Requires fasting conditions, drug interference
**Autophagy assessment** **(LC3, Beclin-1)**	Western blot, flow cytometry, confocal microscopy	Insight into cancer cell treatment susceptibility Western Blot: Sensitivity: high for detecting specific autophagy-related proteins (e.g., LC3-II, p62); Specificity: good, depends on antibody quality Flow Cytometry: Sensitivity: high, can quantify autophagy markers at single-cell level; Specificity: good, antibody-dependent; possible background signal Confocal Microscopy: Sensitivity: high spatial resolution, allows visualization of autophagosome formation; Specificity: very good with specific fluorescent markers (e.g., GFP-LC3)	Complex interpretation in cancer context Western Blot: provides bulk protein levels, no spatial or single-cell resolution, semi-quantitative Flow Cytometry: requires cell suspension, limited spatial information, may need autophagy-specific dyes or tagged proteins Confocal Microscopy: qualitative or semi-quantitative, labor intensive, limited throughput In summary: Western blot gives overall protein expression but lacks spatial/single-cell data; flow cytometry allows quantification in single cells but lacks imaging context; confocal microscopy provides detailed spatial and morphological info but is less quantitative and lower throughput
**Gut microbiome analysis**	16S rRNA sequencing, metagenomics	Influence on immune response and metabolism	Environmental variability, need for bioinformatics
**Immunological tests** **(CD8+, NK, MDSC)**	Flow cytometry, cytotoxicity assays	Evaluation of antitumor immune activity Flow Cytometry: Sensitivity: very high; allows precise identification and quantification of immune cell subsets (CD8+, NK, MDSC); Specificity: excellent, using multiple fluorochrome-conjugated antibodies targeting specific surface/intracellular markers Cytotoxicity Assays: Sensitivity: variable; depends on assay type (e.g., chromium release, LDH release, flow-based killing assays); Specificity: functional assay measuring cell killing but does not identify phenotypes unless combined with flow cytometry	High complexity and cost Flow Cytometry: provides phenotypic and functional markers (e.g., activation, cytokine production) but does not measure direct killing function Cytotoxicity Assays: bulk population measurement, often endpoint assay, can be labor intensive and less quantitative in mixed populations In summary: Flow cytometry excels at detailed phenotypic and functional profiling of immune cells at single-cell resolution, while cytotoxicity assays measure the actual killing capacity but provide less detailed phenotypic information and are generally bulk assays
**Imaging (PET, MRI)**	PET-CT (18F-FDG), DCE-MRI, DWI	Assessment of metabolic activity and tumor morphology *PET:* Sensitivity: very high for detecting metabolic/functional changes at molecular level; Specificity: depends on tracer used; can target specific molecules or processes (e.g., inflammation, metabolism) *MRI:* Sensitivity: high for anatomical detail and tissue contrast; Specificity: good for structural abnormalities; limited molecular specificity without contrast agents *DWI:* Sensitivity: high for detecting changes in water molecule diffusion, useful in acute pathology (e.g., stroke, tumors); Specificity: moderate; changes in diffusion can reflect various pathological processes, not always specific	Expensive, needs advanced equipment, risk of false positives *PET:* limited spatial resolution, exposure to radioactive tracers, high cost *MRI:* longer scan times, contraindications in patients with metal implants, lower sensitivity to molecular changes *DWI:* susceptibility to artifacts, limited molecular specificity, interpretation requires clinical context *In summary:* PET excels in functional and molecular imaging but with lower spatial resolution and radioactivity concerns; MRI provides detailed anatomical imaging; DWI adds functional insight into tissue microstructure but with limited specificity

IL-6—Interleukin-6, CRP—C-reactive Protein, TNF-α—Tumor Necrosis Factor alpha, ELISA—Enzyme-Linked Immunosorbent Assay, IGF-1—Insulin-like Growth Factor 1, IGFBP-3—Insulin-like Growth Factor Binding Protein 3, OGTT—Oral Glucose Tolerance Test, HOMA-IR—Homeostasis Model Assessment of Insulin Resistance, LC3—Microtubule-Associated Protein 1 Light Chain 3, CD8^+^—CD8-positive T lymphocyte (Cytotoxic T cell), NK—Natural Killer Cell, MDSC—Myeloid-Derived Suppressor Cell, PET—Positron Emission Tomography, MRI—Magnetic Resonance Imaging, PET-CT—Positron Emission Tomography–Computed Tomography, 18F-FDG—Fluorodeoxyglucose labeled with Fluorine-18, DCE-MRI—Dynamic Contrast-Enhanced Magnetic Resonance Imagin, DWI—Diffusion-Weighted Imaging.

**Table 3:** Potential benefits, risks and applications of IF in oncology—summary.

Area	Potential Benefits of IF	Possible Risks/Limitations	Research Status
**Supportive therapy**	Reduction in chemotherapy-induced side effects	Risk of malnutrition, particularly in cachectic patients	Preclinical and early-phase clinical studies
**Metabolic impact**	Lower glucose and insulin levels; reduced IGF-1	Risk of hypoglycemia in diabetic patients	Moderate-quality evidence
**Immuno-modulation**	Potential activation of autophagy; enhanced immune response	Limited data in immunosuppressed individuals	Mostly animal studies; limited RCTs
**Microbiota modulation**	Altered gut microbiome composition; improved intestinal barrier	High interindividual variability	Preliminary human data
**Inflammation reduction**	Decreased CRP, TNF-α, IL-6	Requires long-term monitoring and validation	Promising early-stage findings
**Antitumor potential**	Reduced tumor cell proliferation in experimental models	Lack of validation in large-scale human trials	Predominantly preclinical data
**Cancer prevention**	Reduction in metabolic risk factors	Requires prolonged follow-up and population-level studies	Observational evidence; inconclusive in humans

IGF-1—Insulin-like Growth Factor 1, CRP—C-reactive Protein, TNF-α—Tumor Necrosis Factor alpha, IL-6—Interleukin 6, RCTs—Randomized Controlled Trials.

## Data Availability

Not applicable.

## Abbreviations

The following abbreviations are used in this manuscript:

18F-FDG PET	18F-fluorodeoxyglucose positron emission tomography
ANC	absolute neutrophil count
CBC	Complete blood count
CCL2	chemokine (C-C motif) ligand 2
CRP	C-reactive protein
CTLA-4	cytotoxic T-lymphocyte antigen 4
DSR	differential stress resistance
DWI	diffusion-weighted imaging
ELISA	Enzyme-Linked Immunosorbent Assay
FMD	fasting-mimicking diets
G-CSF	granulocyte colony-stimulating factor
GLUT1	glucose transporter type 1
HDL	high-density lipoprotein
HLA-DR^−^	Human Leukocyte Antigen—DR isotype/low
HOMA-IR	homeostatic model assessment for insulin resistance
IF	Intermittent fasting
IFN-γ	interferon gamma
IGF-1	insulin-like growth factor 1
IGFBP-3	Insulin-like Growth Factor Binding Protein 3
IL-6	interleukin-6
LAG3	lymphocyte activation gene-3
LC3-II	microtubule-associated protein 1 light chain 3
LDL	low-density lipoprotein
MCV	Mean Corpuscular Volume
MDSCs	myeloid-derived suppressor cells
MRI	magnetic resonance imaging
NK	natural killer (cells)
Nrf2	nuclear factor erythroid 2-related factor 2
OGTT	oral glucose tolerance tests
PD-1	programmed cell death protein 1
PMN-MDSC	Polymorphonuclear MDSCs
RBC	red blood cells
RDW	red cell distribution width
ROS	reactive oxygen species
SCFAs	short-chain fatty acids
TNF-α	tumor necrosis factor alpha
WBC	white blood cells

## References

[1] Siegel R.L., Miller K.D., Wagle N.S., Jemal A. (2024). Cancer Statistics, 2024. CA Cancer J. Clin..

[2] Tinsley G.M., La Bounty P.M. (2015). Effects of Intermittent Fasting on Body Composition and Clinical Health Markers in Humans. Nutr. Rev..

[3] Sutton E.F., Beyl R., Early K.S., Cefalu W.T., Ravussin E., Peterson C.M. (2018). Early Time-Restricted Feeding Improves Insulin Sensitivity, Blood Pressure, and Oxidative Stress Even without Weight Loss in Men with Prediabetes. Cell Metab..

[4] Safdie F.M., Dorff T., Quinn D., Fontana L., Wei M., Lee C., Cohen P., Longo V.D. (2009). Fasting and Cancer Treatment in Humans: A Case Series Report. Aging.

[5] Raffaghello L., Lee C., Safdie F.M., Wei M., Madia F., Bianchi G., Longo V.D. (2008). Starvation-Dependent Differential Stress Resistance Protects Normal but Not Cancer Cells against High-Dose Chemotherapy. Proc. Natl. Acad. Sci. USA.

[6] Aktas A., Thomas S., Barrett M., Sui J. (2025). Nutritional Interventions in Advanced Cancer: A Scoping Review. Am. J. Hosp. Palliat. Med..

[7] Fatima G., Mehdi A.A., Fedacko J., Hadi N. (2025). Fasting as Cancer Treatment: Myth or Breakthrough in Oncology. Cureus.

[8] Longo V.D., Panda S. (2016). Fasting, Circadian Rhythms, and Time-Restricted Feeding in Healthy Lifespan. Cell Metab..

[9] Lee C., Raffaghello L., Brandhorst S., Safdie F.M., Bianchi G., Martin-Montalvo A., Pistoia V., Wei M., Hwang S., Merlino A. (2012). Fasting Cycles Retard Growth of Tumours and Sensitize a Range of Cancer Cell Types to Chemotherapy. Sci. Transl. Med..

[10] Antunes F., Erustes A.G., Costa A.J., Nascimento A.C., Bincoletto C., Ureshino R.P., Pereira G.J.S., Smaili S.S. (2018). Autophagy and Intermittent Fasting: The Connection for Cancer Therapy?. Clinics.

[11] Bahrami A., Haghighi S., Moghani M.M., Khodakarim N., Hejazi E. (2024). Fasting mimicking diet during neo-adjuvant chemotherapy in breast cancer patients: A randomized controlled trial study. Front. Nutr..

[12] Di Biase S., Lee C., Brandhorst S., Manes B., Buono R., Cheng C.W., Cacciottolo M., Martin-Montalvo A., de Cabo R., Wei M. (2016). Fasting-Mimicking Diet Reduces HO-1 to Enhance T Cell-Mediated Tumour Cytotoxicity. Cancer Cell.

[13] Kalam F., James D.L., Li Y.R., Coleman M.F., Kiesel V.A., Cespedes Feliciano E.M., Hursting S.D., Sears D.D., Kleckner A.S. (2023). Intermittent Fasting Interventions to Leverage Metabolic and Circadian Mechanisms for Cancer Treatment and Supportive Care Outcomes. J. Natl. Cancer Inst. Monogr..

[14] Dorff T.B., Groshen S., Garcia A., Shah M., Tsao-Wei D., Pham H., Cheng C.-W., Brandhorst S., Cohen P., Wei M. (2016). Safety and feasibility of fasting in combination with platinum-based chemotherapy. BMC Cancer.

[15] de Groot S., Lugtenberg R.T., Cohen D., Welters M.J.P., Ehsan I., Vreeswijk M.P.G., Smit V.T.H.B.M., de Graaf H., Heijns J.B., Portielje J.E.A. (2020). Fasting mimicking diet as an adjunct to neoadjuvant chemotherapy for breast cancer in the multicentre randomized phase 2 DIRECT trial. Nat. Commun..

[16] de Groot S., Pijl H., van der Hoeven J.J.M., Kroep J.R. (2020). Effects of Short-Term Fasting on Cancer Treatment. J. Exp. Clin. Cancer Res..

[17] Vernieri C., Fucà G., Ligorio F., Huber V., Vingiani A., Iannelli F., Raimondi A., Rinchai D., Frigè G., Belfiore A. (2022). Fasting-mimicking diet is safe and reshapes metabolism and antitumour immunity in patients with cancer. Cancer Discov..

[18] Fu C., Lu Y., Zhang Y., Yu M., Ma S., Lyu S. (2021). Intermittent fasting suppressed splenic CD205+ G-MDSC accumulation in a murine breast cancer model by attenuating cell trafficking and inducing apoptosis. Food Sci. Nutr..

[19] Zhong Z., Zhang H., Nan K., Zhong J., Wu Q., Lu L., Yue Y., Zhang Z., Guo M., Wang Z. (2023). Fasting-Mimicking Diet Drives Antitumour Immunity against Colorectal Cancer by Reducing IgA-Producing Cells. Cancer Res..

[20] Luo M., Wang Q., Sun Y., Zhang J., Li Y., Chen L., Liu X., Liu J., Zhang Y., Li X. (2024). Fasting-mimicking diet remodels gut microbiota and suppresses colorectal cancer progression. NPJ Biofilms Microbiomes.

[21] Oliveira M.L., Biggers A., Oddo V.M., Naylor K.B., Chen Z., Hamm A., Pezley L., Bernabé B.P., Gabel K., Sharp L.K. (2024). Design of a remote time-restricted eating and mindfulness intervention to reduce risk factors associated with early-onset colorectal cancer development among young adults. Nutrients.

[22] Gamal El-Tahawy N.F., Ahmed Rifaai R. (2023). Intermittent Fasting Protects Against Age-Induced Rat Benign Prostatic Hyperplasia via Preservation of Prostatic Histomorphology, Modification of Oxidative Stress, and Beclin-1/P62 Pathway. Microsc. Microanal..

[23] Minciuna I., van Kleef L.A., Stefanescu H., Procopet B. (2022). Is Fasting Good When One Is at Risk of Liver Cancer?. Cancers.

[24] Wilhelmi de Toledo F., Grundler F., Sirtori C.R., Ruscica M. (2020). Unravelling the Health Effects of Fasting: A Long Road from Obesity Treatment to Healthy Life Span Increase and Improved Cognition. Ann. Med..

[25] de Cabo R., Mattson M.P. (2019). Effects of Intermittent Fasting on Health, Aging, and Disease. N. Engl. J. Med..

[26] Weng M., Chen W., Chen X., Lu H., Sun Z., Yu Q., Sun P., Xu Y., Zhu M., Jiang N. (2020). Fasting Inhibits Aerobic Glycolysis and Proliferation in Colorectal Cancer via the Fdft1-Mediated AKT/mTOR/HIF1α Pathway Suppression. Nat. Commun..

[27] Maecker H.T., McCoy J.P., Nussenblatt R. (2012). Standardizing Immunophenotyping for the Human Immunology Project. Nat. Rev. Immunol..

[28] Chen J., Su R., He Y., Chen J. (2025). Intermittent fasting inhibits the development of colorectal cancer in APC^Min^/+ mice through gut microbiota and its related metabolites. Front. Microbiol..

[29] Gopalakrishnan V., Spencer C.N., Nezi L., Reuben A., Andrews M.C., Karpinets T.V., Prieto P.A., Vicente D., Hoffman K., Wei S.C. (2018). Gut Microbiome Modulates Response to Anti–PD-1 Immunotherapy in Melanoma Patients. Science.

[30] Yang Y.F., Li C.H., Cai H.Y., Lin B.S., Kim C.H., Chen H.C. (2022). Application of Metabolic Reprogramming to Cancer Imaging and Diagnosis. Int. J. Mol. Sci..

[31] Just N. (2014). Improving tumour heterogeneity MRI assessment with histograms. Br. J. Cancer.

[32] Wunderle C., Martin E., Wittig A., Tribolet P., Lutz T.A., Köster-Hegmann C., Stanga Z., Mueller B., Schuetz P. (2025). Comparison of the inflammatory biomarkers IL-6, TNF-α, and CRP to predict the effect of nutritional therapy on mortality in medical patients at risk of malnutrition: A secondary analysis of the EFFORT randomized clinical trial. J. Inflamm..

[33] Zalfa C., Paust S. (2021). Natural Killer Cell Interactions With Myeloid Derived Suppressor Cells in the Tumor Microenvironment and Implications for Cancer Immunotherapy. Front. Immunol..

[34] Maifeld A., Bartolomaeus H., Löber U., Avery E.G., Steckhan N., Markó L., Wilck N., Hamad I., Šušnjar U., Mähler A. (2021). Fasting Alters the Gut Microbiome Reducing Blood Pressure and Body Weight in Metabolic Syndrome Patients. Nat. Commun..

[35] Pollak M. (2012). The insulin and insulin-like growth factor receptor family in neoplasia: An update. Nat. Rev. Cancer.

[36] Giovannucci E., Harlan D.M., Archer M.C., Bergenstal R.M., Gapstur S.M., Habel L.A., Pollak M., Regensteiner J.G., Yee D. (2010). Diabetes and cancer: A consensus report. Diabetes Care.

[37] Brandhorst S., Choi I.Y., Wei M., Longo V.D. (2015). A Periodic Diet that Mimics Fasting Promotes Multi-System Regeneration, Enhanced Cognitive Performance, and Healthspan. Cell Metab..

[38] Wilhelmi de Toledo F., Grundler F., Goutzourelas N., Tekos F., Vassi E., Mesnage R., Kouretas D. (2020). Influence of Long-Term Fasting on Blood Redox Status in Humans. Antioxidants..

[39] Jia H., Wei J., Zheng W., Zhang L., Wang Q., Liu M. (2025). The Dual Role of Autophagy in Cancer Stem Cells: Implications for Tumour Progression and Therapy Resistance. J. Transl. Med..

[40] Mahri S., Villa R., Shiau Y.P., Tang M., Racacho K.J. (2025). Nanomedicine Approaches for Autophagy Modulation in Cancer Therapy. Small Methods.

[41] Lee J., Cheong H. (2025). The Role of A20 in Cancer: Friend or Foe?. Cells.

[42] Alirezaei M., Kemball C.C., Flynn C.T., Wood M.R., Whitton J.L., Kiosses W.B. (2010). Short-Term Fasting Induces Profound Neuronal Autophagy. Autophagy.

[43] Madeo F., Zimmermann A., Maiuri M.C., Kroemer G. (2015). Essential Role for Autophagy in Life Span Extension. J. Clin. Investig..

[44] Pietrocola F., Pol J., Vacchelli E., Rao S., Enot D.P., Baracco E.E., Levesque S., Castoldi F., Jacquelot N., Yamazaki T. (2017). Caloric Restriction Mimetics Enhance Anticancer Immunosurveillance. Cancer Cell.

[45] Bortnik S., Gorski S.M. (2017). Clinical Applications of Autophagy Proteins in Cancer: From Potential Targets to Biomarkers. Int. J. Mol. Sci..

[46] Klionsky D.J., Abdel-Aziz A.K., Abdelfatah S., Abdellatif M., Abdoli A., Abel S., Abeliovich H., Abildgaard M.H., Abudu Y.P., Acevedo-Arozena A. (2021). Guidelines for the Use and Interpretation of Assays for Monitoring Autophagy (4th Edition). Autophagy.

[47] Udumula M.P., Singh H., Rashid F., Poisson L., Tiwari N., Dimitrova I., Hijaz M., Gogoi R., Swenor M., Munkarah A. (2023). Intermittent Fasting Induced Ketogenesis Inhibits Mouse Epithelial Ovarian Cancer by Promoting Antitumour T Cell Response. iScience.

[48] Cossarizza A., Chang H.-D., Radbruch A., Akdis M., Andrä I., Annunziato F., Ballesteros J., Barnaba V., Battistini L., Bauer W. (2019). Guidelines for the Use of Flow Cytometry and Cell Sorting in Immunological Studies (Second Edition). Eur. J. Immunol..

[49] Alter G., Malenfant J.M., Altfeld M. (2004). CD107a as a Functional Marker for the Identification of Natural Killer Cell Activity. J. Immunol. Methods.

[50] Leng S.X., McElhaney J.E., Walston J.D., Xie D., Fedarko N.S., Kuchel G.A. (2008). ELISA and Multiplex Technologies for Cytokine Measurement in Inflammation and Aging Research. J. Gerontol. A Biol. Sci. Med. Sci..

[51] Grivennikov S.I., Greten F.R., Karin M. (2010). Immunity, Inflammation, and Cancer. Cell.

[52] Cienfuegos S., Gabel K., Kalam F., Ezpeleta M., Lin S., Varady K.A. (2023). Effects of Intermittent Fasting and Caloric Restriction on Inflammatory Biomarkers: A Systematic Review and Meta-Analysis of Randomized Controlled Trials. Obes. Rev..

[53] Scheer F.A.J.L., Hilton M.F., Mantzoros C.S., Shea S.A. (2014). Endogenous Circadian Regulation of Pro-Inflammatory Cytokines and Chemokines in the Presence of Bacterial Lipopolysaccharide in Humans. Brain Behav. Immun..

[54] Pepys M.B., Hirschfield G.M. (2003). C-Reactive Protein: A Critical Update. J. Clin. Investig..

[55] Nencioni A., Caffa I., Cortellino S., Longo V.D. (2018). Fasting and Cancer: Molecular Mechanisms and Clinical Application. Nat. Rev. Cancer.

[56] Pollak M. (2008). Insulin and Insulin-Like Growth Factor Signalling in Neoplasia. Nat. Rev. Cancer.

[57] Renehan A.G., Zwahlen M., Minder C., O’Dwyer S.T., Shalet S.M., Egger M. (2004). Insulin-Like Growth Factor (IGF)-I, IGF Binding Protein-3, and Cancer Risk: Systematic Review and Meta-Regression Analysis. Lancet.

[58] Zhao X., Yang J., Huang R., Guo M., Zhou Y., Xu L. (2021). The role and its mechanism of intermittent fasting in tumors: Friend or foe?. Cancer Biol. Med..

[59] Frystyk J., Delhanty P.J.D., Skjærbæk C., Baxter R.C. (1999). Changes in the circulating IGF system during short-term fasting and refeeding in rats. Am. J. Physiol. Endocrinol. Metab..

[60] Clemmons D.R. (2011). on behalf of the conference participants. Consensus Statement on the Standardization and Evaluation of Growth Hormone and Insulin-Like Growth Factor Assays. Clin. Chem..

[61] Liu Y., Zhou Q., Song S., Tang S. (2021). Integrating Metabolic Reprogramming and Metabolic Imaging to Predict Breast Cancer Therapeutic Responses. Trends Endocrinol. Metab..

